# Meta-analysis of behavioral treatments for selective mutism: findings from selective mutism questionnaire (SMQ) and school speech questionnaire (SSQ)

**DOI:** 10.1186/s13034-025-00891-8

**Published:** 2025-04-03

**Authors:** Daichi Iimura, Natsuki Tsujita, Mizuki Aoki, Hiromichi Hagihara

**Affiliations:** 1 Institute of Human Sciences, University of Tsukuba, 1-1-1 Tennodai, Tsukuba- shi, Ibaraki 305-8572 Japan; 2 Institute for Transdisciplinary Graduate Degree Programs, Osaka University, Osaka, Japan; 3 Center for the Study of Co* Design, Osaka University, Osaka, Japan; 4 Graduate School of Comprehensive Human Sciences, University of Tsukuba, Ibaraki, Japan; 5 Research Fellow of the Japan Society for the Promotion of Science, Tokyo, Japan; 6 Graduate School of Human Sciences, Osaka University, Osaka, Japan

**Keywords:** Selective mutism, Behavioral treatment, Selective mutism questionnaire (SMQ), School speech questionnaire (SSQ), Meta-analysis

## Abstract

**Background:**

This meta-analysis aims to assess the efficacy of behavioral therapy for selective mutism (SM) using the Selective Mutism Questionnaire (SMQ) and School Speech Questionnaire (SSQ).

**Methods:**

We analyzed 12 articles involving 472 participants and comprising three randomized controlled trials and nine before-after or multiple baseline study designs.

**Results:**

Our findings indicate a significant improvement in symptoms of SM, as indicated by the SMQ total, the SMQ subsections scores (school, home, and public), and the SSQ. The mean SMQ total score improved by 0.51 [95% confidence interval of 0.32–0.70] with a large effect size (Hedges’ adjusted g = 1.00 [0.62–1.39]). This effect did not differ significantly based on the study design. Based on the treatment strategy, web-based treatment demonstrated less improvement (0.12 [− 0.11–0.36]) compared with face-to-face treatment (0.59 [0.39–0.80]).

**Conclusions:**

This meta-analysis revealed that SM treatment significantly improved their speaking behavior measured by the SMQ and SSQ, though potential subgroups that influence the treatment efficacy remain. SMQ has also shown validity and responsiveness as an outcome tool for behavioral therapy for SM. Further clinical practices with randomized controlled trials are recommended to clarify the potential differences of treatment or target populations.

**Supplementary Information:**

The online version contains supplementary material available at 10.1186/s13034-025-00891-8.

## Key points

- Estimating the effect size of selective mutism (SM) treatment is essential to provide evidence-based intervention for children with SM.
- The Selective Mutism Questionnaire (SMQ) and School Speech Questionnaire (SSQ) are commonly used to assess children with SM.
- We conducted a meta-analysis to assess the efficacy of behavioral SM treatment using the SMQ and SSQ, analyzing 12 articles with 472 participants.
- SM treatment significantly improved the weighted mean difference scores on both the SMQ and SSQ, with face-to-face treatment being more effective than web-based treatment.

**Supplementary Information:**

The online version contains supplementary material available at 10.1186/s13034-025-00891-8.

## Background

The Diagnostic and Statistical Manual of Mental Disorders (DSM-5) describes selective mutism (SM) as an anxiety disorder characterized by the consistent inability to speak in certain social situations where speech is expected, such as school [1]. The prevalence is reported to range from 0.03 to 1%, and the age of onset is usually younger than five years [1]. According to Muris and Ollendick [2], various psychosocial approaches for SM, such as psychodynamic and systems approaches, have been applied along with cognitive behavioral therapy (CBT). Specifically, CBT has been particularly effective [2, 3] and almost all interventions in recent studies include behavioral strategies such as reinforcement, exposure, and cognitive restructuring [2]. Given that the central element of the approach is behavior, it is important to assess the speaking behavior of children with SM appropriately.

Measuring treatment outcomes is crucial for verifying the effectiveness of SM treatment. It helps to determine whether the treatment is working and provides valuable insights for further improvement. Rodrigues Pereira et al. [4] reviewed 91 articles from 2010 to 2021 on the assessment and treatment of children with SM. They found that the most used questionnaire was the Selective Mutism Questionnaire (SMQ; Bergman, Keller, Piacentini, & Bergman [5]), which was used in 32 articles (35.2%), followed by the School Speech Questionnaire (SSQ; Bergman, Piacentini, & McCracken [6]), which was used in 11 articles (12.1%). The SMQ evaluates children’s speech frequency in several situations, as reported by their parents [5], and The SSQ assesses children’s frequency of speech, especially at school, as reported by their teachers [6, 7]. The SMQ and SSQ are standardized in many languages, are widely adapted, and have been shown to effectively evaluate treatment changes in children with SM [7].

Several meta-analyses have been published on SM treatment (e.g., Steains, Malouff, & Schutte [8]; Hipolito et al. [9]. However, their applicability in clinical settings is limited. First, they included outcome measures other than the SMQ, such as the Strong Narrative Assessment Procedure (SNAP; Steains et al. [8]; Strong, Mayer, & Mayer [10]), or only reported the total score of the SMQ [9]. The lack of consistency of the outcome measures resulted in tentative conclusions about the synthesis of results [9]. Second, these meta-analyses strictly limited the articles on which they focused to randomized controlled trial (RCT) designs, resulting in small sample sizes. Østergaard [3] pointed out the limited amount of literature that used an RCT design in their systematic review. Thus, it is difficult to estimate the treatment effect size of specific outcomes, i.e., SMQ and SSQ. Therefore, this study aimed to evaluate the efficacy of behavioral interventions that used the SMQ or SSQ as a clinical outcome by conducting a meta-analysis of behavioral intervention studies that include various study designs. We provide the standardized effect size of SMQ and SSQ as well as non-standardized values that are easy to interpret for clinical practice. Furthermore, in some studies, interventions have been associated with improvements in SM symptoms [11, 12], while in others, no such improvements were observed [13, 14]. To elucidate potential reasons for this discrepancy, this study also investigated whether study designs or intervention strategies influence the changes in the overall score of the SMQ. This will also facilitate the assessment of the extent of improvement in future treatments.

## Methods

### Literature search strategy

Based on the Preferred Reporting Items for Systematic Reviews and Meta-Analysis (PRISMA) 2020 guidelines [15], we utilized three information sources: (a) a previous review [4], (b) multi-database searches, and (c) manual literature searches. (a) We extracted SMQ data from the review by Rodrigues Pereira et al. [4], whose search covered articles from 2010 to July 2021. (b) We used Web of Science, PubMed, PsychInfo, Medline, Education Resources Information Center (ERIC), and Cochrane to identify eligible references. The search was conducted on October 14, 2022 and updated on February 17, 2025, by searching for articles containing the word “selective mutism” across databases from 2021 to February 2025 to cover articles that were published later than Rodrigues Pereira et al.’s [4] search. Because of the narrow range of publication year in the database search, only “selective mutism” was used as the search term, and all relevant literature was included in the screening process. We neither used MeSH or database-specific indexing terms nor applied search options such as specific language or study design. (c) Manual literature searches were conducted using reference lists of review articles, relevant publications, published books, and searches on other databases (i.e., Google Scholar). The search results were exported into a reference management software (Endnote X9.3.3 for Windows), and duplicates were manually removed.

### Inclusion and exclusion criteria

The eligibility criteria were as follows: the articles had to (a) be SM studies irrespective of the participants’ ages, (b) involve behavioral intervention for SM as the primary aim, and (c) evaluate either total SMQ scores or SMQ subsections in pre- and post-treatment. To include articles that were not gray literature, involved behavioral interventions for participants whose primary concern was SM, and assessed SMQ scores statistically, we excluded articles if (a) they were conference papers, reviews, commentaries, or special articles that did not describe a specific case, (b) they were dissertations, (c) participants did not show characteristics or symptoms of SM at the time of intervention, (d) other neurological diseases or mental disorders could explain participants’ characteristics or symptoms, (e) SMQ was not used as a clinical outcome, (f) they included a pharmacological or medical treatment, (g) SM intervention was not their primary aim, and (h) no statistical values were reported (i.e., sample size and mean and standard deviation of the outcome).

### Study selection

Articles were included for analysis after a two-stage process following the PRISMA 2020 flow diagram [15]: screening the title and abstract (Stage 1 screening) and screening the full text of the article (Stage 2 screening). In Stage 1 screening, the title and abstracts of all articles obtained through the databases were screened based on the eligibility and exclusion criteria. In addition, articles with unclear eligibility were also moved forward to Stage 2 screening for detailed screening. In Stage 2 screening, the full text of each article was examined closely to determine if it met the eligibility criteria. The first (DI) and second (NT) authors evaluated each article independently, and Cohen’s kappa was calculated to assess the reliability of the authors’ screening in both stages. Disagreements or doubts regarding a particular article at either stage were resolved through discussion.

### SMQ and SSQ

The SMQ is a 17-item parent-rated questionnaire. It uses a four-point Likert scale that ranges from *neve*r (0) to *always* (3) to evaluate the frequency of speaking behavior in three different settings: school (six items), home (six items), and public (five items). The Cronbach’s α coefficients for internal consistency were 0.97, 0.97, 0.88, and 0.96 for the total scale, school, home, and public subsections, respectively [5]. The SSQ is a scale evaluated by teachers using a 4-point Likert scale, similar to the SMQ, comprising six items in the school situation [6, 7]. It has an internal consistency of 0.76, based on Cronbach’s α coefficient [11].

Scores on the SMQ and SSQ range from 0 to 51 and 0–18, respectively. Lower scores indicate more severe symptoms of SM. Because each subsection of the SMQ contains an unequal number of items, comparing scores across subsections is difficult; therefore, the average score for each subsection was calculated by dividing its total scores by the number of items.

###  Data extraction

The total pre- and post-treatment SMQ scores were extracted from each article as the primary outcome, along with the sample size. The scores of three subsections of the SMQ (school, home, and public) and the SSQ were also extracted. If an article compared two groups, such as treatment and control groups, the pre- and post-treatment score differences in the treatment group were analyzed. If a control group later received the same treatment as the treatment group, we combined both groups’ participants in the meta-analysis. The control group reported no changes in SMQ scores previously [7, 11]; therefore, the SMQ scores of the control group were excluded in our meta-analysis.

To summarize the articles, each was coded across the following dimensions: author(s) and year of publication, study design, sample size, participant’s primary information such as age and gender distribution, intervention program, outcome measured by psychological scales, and follow-up information. Two authors (DI and NT) coded this information, and all authors confirmed the coding process. Any disagreements were resolved through discussion among all authors.

### Risk of bias assessment

As our inclusion criteria included all study designs such as RCT or before-after design studies, we used two assessment tools for the Risk of Bias (RoB) evaluation. Cochrane criteria [16] were used in the RCT design, and the Risk of Bias Assessment Tool for Nonrandomised Studies (RoBANS [17]) was used in the non-randomized study design; thus, these two tools are compatible. The RoB in RCTs was assessed as high, low, or unclear based on criteria including random sequence generation, allocation concealment, blinding for participants and personnel, blinding for outcome assessor, incomplete outcome data, and selective outcome reporting in Cochrane criteria [16]. Similarly, articles with a non-randomized design were assessed for RoB based on criteria including selection of participants, confounding variables, measurement of exposure, blinding of outcome assessment, incomplete outcome data, and selective outcome reporting in RoBANS [17]. RoBANS covers virtually all study designs except for RCTs for RoB assessment [17]. Articles with higher rigor were associated with lower RoB and produced results closer to the true effect [16]. Two authors (NT and MA) independently evaluated the six subsections for each article, and any disagreements were resolved through discussions among three authors (DI, NT, and MA) to reach a consensus.

### Statistical analysis

We synthesized the SMQ and SSQ scores for analysis. Quantitative analysis was conducted using a random-effects model to estimate the weighted mean difference scores and 95% confidence intervals (CI) between the pre- and post-treatment of each outcome. As the standardized mean difference, Hedges’ adjusted g was also calculated to facilitate comparison with previous or future findings of other meta-analyses. The analyses were performed for each outcome, that is, total SMQ, SMQ subsection, and SSQ scores. Articles or data that contained missing values, such as mean, standard deviation, and sample size in pre- and post-treatment, for which meta-analysis could not be performed, were excluded from the analyses. Statistical analysis and visualization were performed using Review Manager, version 5.4.1 [18].

To assess the impact of statistical heterogeneity, we used Cochrane’s Q and I^2^ statistics in line with a previous meta-analysis [8]. Cochrane’s Q tests whether effect sizes vary significantly among studies using a chi-square test. As Cochrane’s Q may be less sensitive in detecting heterogeneity in small sample sizes, we also used I^2^, which represents the percentage of variance in an outcome that reflects a real difference regardless of the sample size [19]. High heterogeneity, e.g., > 75%, suggests the existence of subgroups within the included studies. Sensitivity analyses were performed and compared between subgroups to assess the potential subgroup difference. The comparison groups were pre-specified based on study design (i.e., RCTs or non-RCT), sample size, intervention strategy (i.e., face-to-face or web-based), or treatment period. The cut-off points for sample size and treatment period variables were determined based on their distribution after data extraction. We compared the outcomes between the two groups to examine whether these subgroups affected the weighted mean difference scores of the total SMQ. Further, to investigate the long-term treatment effect after the SM intervention, we included follow-up data and calculated the weighted mean difference scores between periods of pre-treatment and follow-up.

We confirmed the efficacy of SM treatment by examining whether the 95% CI of the weighted mean difference scores of the SMQ total overlapped zero. If the 95% CI overlaps zero, representing the null effect of the treatment efficacy, a treatment’s effectiveness is interpreted as imprecise.

Publication bias, which refers to the tendency for published papers to predominantly report favorable treatment outcomes, was investigated by visualizing a funnel plot and conducting statistical analyses using software for statistical computing (R 4.0.4.; R Core Team [20]).

**Fig. 1:**
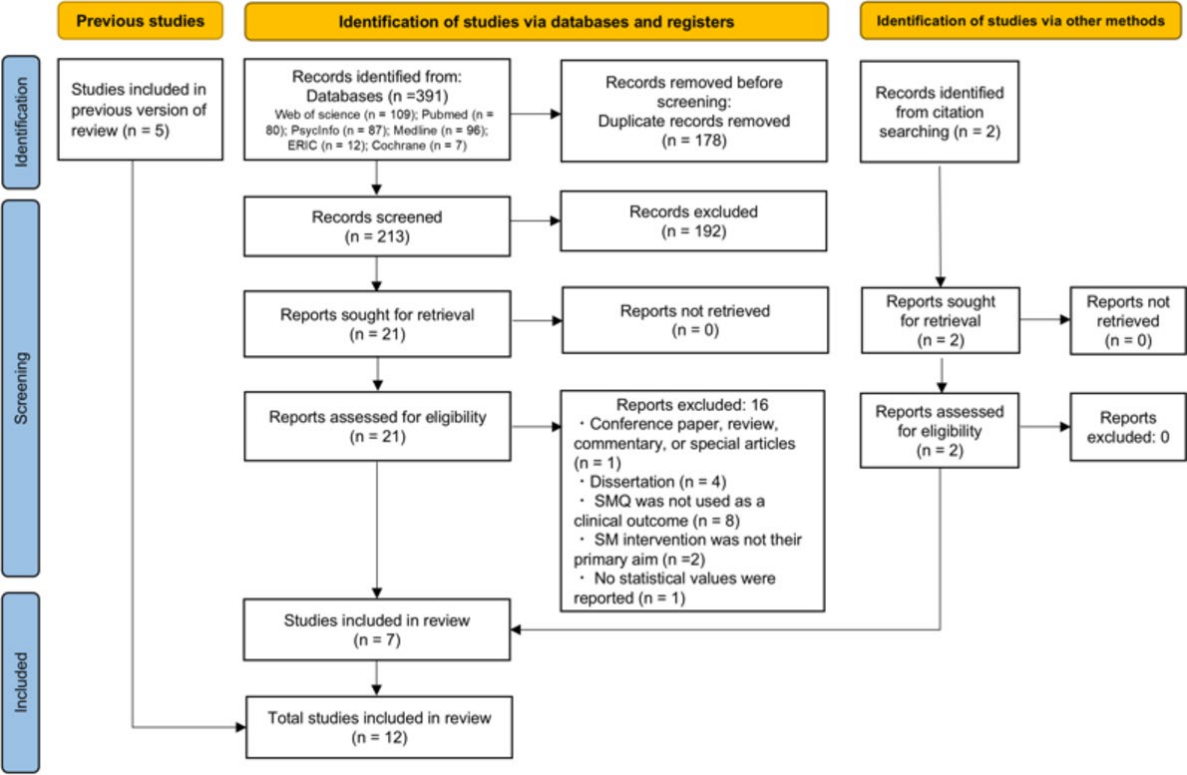
PRISMA 2020 flow diagram

## Results

### Article search process

The article search process is shown in Fig. 1. Of the 56 articles from the previous review [4], five [11, 21–24] were eligible for our meta-analysis. An electronic database search yielded 391 citations. After manually excluding articles with duplicate indexing, 213 were selected for Stage 1 screening. Based on eligibility criteria, 192 articles were excluded from Stage 1 screening. Cohen’s kappa was 0.75, indicating substantial agreement between the two authors [25]. Any articles with discrepancies advanced to Stage 2 screening, and the full texts of the 21 articles were examined. Sixteen studies were excluded based on the exclusion criteria with Cohen’s kappa of 1.00, demonstrating complete agreement [25]. A list of the excluded studies and the reasons for their exclusion are displayed in the appendix. Finally, five articles [14, 26–29] were considered eligible. In addition, two articles [13, 30] were identified through a manual citation search using Google Scholar. Both articles met the eligibility criteria for inclusion in this study. Finally, 12 articles (five from a previous review, five from database searches, and two from manual citation searching), with a total of 472 participants, were included in this review.

###  Summary of the included articles

To illustrate article information synthesized in our meta-analysis, summaries of the articles are provided in Table 1. Three articles [11, 13, 22] employed the RCT design, eight [14, 21, 23, 24, 26–28, 30] employed before-after study designs (note that part of Oerbeck et al.’s [24] study was also reported in other articles [31, 32]), while one [29] adopted the multiple baseline design. Of the three studies with RCT designs, two [11, 13] provided no special treatment to the control group. One study [22] provided the same treatment to the control group later, and participants in both groups were analyzed after confirming that the SMQ score change was comparable (mean difference ≤ 0.1). The sex ratio varied, with some having more girls, some having more boys, and another having an even split. Treatments commonly used included Parent–Child Interaction Therapy (PCIT) or behavior-focused treatments. Additionally, treatment modalities were web-based in three articles [13, 14, 27]. Outcome measures were widely used for SM symptoms (SMQ or SSQ) as well as behavioral problems such as using the Child Behavior Checklist (CBCL; Achenbach & Rescorla [33]), language functioning using SNAP [10], and anxiety using the Spence Children’s Anxiety Scale [34]. Nine out of 12 articles included follow-up assessment post intervention.

**Table 1:** Summary of articles included in the review

Authors (year)	Study design	Sample size	Age		Gender distribution			Intervention program	Outcomes measured	Follow-up
			Mean (SD)	Range	Female	Male	% Female			
Aldrich et al. [30]	Before-After	112^a^	7.26 (2.55)	3–14	71	41^b^	63.4	PCIT-SM	SMQ, SSQ, CALIS	No
Bergman et al. [11]	RCT ^c^	21	5.43 (1.16)	4–8	10	11	47.6	IBSTM	SMQ, SSQ, CGI-I, SASC-R, SNAP	Yes: After three months
		12	5.25 (1.14)		5	7	41.7			
Catchpole et al. [21]	Before-After	31	6.47 (1.68)	4.0–9.75	16	15	51.6	PCIT-SM	SMQ, SSQ, CSQ-8, SCARED, SNAP,	Yes: After three months and one year
Cornacchio et al. [22]	RCT ^d^	29 ^d^	6.6 (1.3)	5–9	22	7	75.9	IGBT	SMQ, CBCL, CSR, CGAS	Yes: After four and eight weeks
Haggerty et al. [26]	Before-After ^e^	25	7.9 (-)	4–11	20	5	80.0	Intensive Summer Day Camp Intervention	SMQ, DBR, SCARED,	Yes: After three months
Hong et al. [27]	Before-After	9	6.1 (1.5)	-	5	4	55.6	Remote IGBT	SMQ, ADIS, CBCL, CGAS, CGI-I, FSSM	Yes: After four months
Klein et al. [23]	Before-After	40	6.78 (1.58)	5–12	25	15	62.5	S-CAT	SMQ, CBCL, TNL	Yes: After six weeks
Kupferberg et al. [28]	Before-After	16 ^f^	13.8 (2.2)	10–17	11	5	68.8	IGBT	SMQ, ADIS, SEQ-C, SM-self report, SSIS-RS	Yes: After two and five months ^g^
Oerbeck et al. [24] ^h^	Before-After	30	6 (unknown)	3–9	20	10	66.7	School-based CBT	SMQ, SSQ	Yes: After one and five year(s)
Ooi et al. [13]	RCT ^c^	21	8.62 (1.99)	6–11	8	13	38.1	Web-based CBT	SMQ, ACAS, CGI-I	No
		10	8.70 (1.77)		6	4	60.0			
Siroky et al. [29]	MBD	5	6.2 (1.5)	4–8	2	3	40.0	IBSTM	SMQ, ADIS, SASC-R, SCARED	No
Tan et al. [14]	Before-After	20	8.45 (1.96)	6–12	9	11	45.0	VRET	SMQ, CBCL, CGAS, CGI-I, SCAS	Yes: After one and three month(s)

*ACAS* Asian Children’s anxiety scale-caretaker version, *ADIS* anxiety diagnostic interview schedule, *CALIS* child anxiety life interference scale, *CBCL* child behavior checklist, *CBT* cognitive behavioral treatment, *CGAS* children’s global assessment scale, *CGI-I* Clinical global impression–improvement scales, *CSQ-8* client satisfaction questionnaire, *CSR* Clinical Severity Rating, *DBR* Daily behavioral report, *FSSM* Frankfurt scale of selective mutism, *IBTSM* integrated behavior therapy for selective mutism, *IGBT* intensive group behavioral treatment, *MBD* multiple baseline design, *PCIT* parent child interaction therapy, *RCT* randomized controlled trial, *S-CAT* social communication anxiety treatment, *SASC-R* social anxiety scale for children-revised, *SCARED* Screen for child anxiety related emotional disorders, *SEQ-C* self-efficacy questionnaire for children, *SMQ* selective mutism questionnaire, *SSQ* school speech questionnaire, *SNAP* strong narrative assessment procedure, *SSIS-RS* social skills improvement system rating scales-student, *TNL* test of narrative language, *VRET* virtual reality exposure therapy

^a^Three participants did not proceed to the treatment

^b^Includes one transgender male

^c^Upper row: all participants. Lower row: participants in the treatment group, i.e., participants analyzed in our meta-analysis

^d^Control group later received treatment and thus included in the meta-analysis

^e^The paper refers to a single-case replicated AB design, but we refer to it as Before-After because it can be regarded as a pre-post design

^f^Four participants had no post-treatment outcome

^g^Because there were no immediate post-treatment outcomes, the first follow-up was considered as the main outcome and the second follow-up as the first follow-up outcome

^h^Part of the same sample was reported by Oerbeck et al. [31, 32]

Table 2 shows the outcomes of the meta-analysis. Four articles [11, 26, 28, 29] did not report the scores of the SMQ subsections, and Cornacchio et al. [22] and Hong et al. [27] did not report the scores of the SMQ-total and SMQ-school subsections. Eight articles [13, 14, 22, 23, 26–29] did not include the SSQ scores.

**Table 2:** Available outcomes of the meta-analysis

Study	SMQ-total	SMQ-School	SMQ-Home	SMQ-Public	SSQ
Aldrich et al. [30]	+	+	+	+	+
Bergman et al. [11]	+	–	–	–	+
Catchpole et al. [21]	+	+	+	+	+
Cornacchio et al. [22]	–	–	+	+	–
Haggerty et al. ^a^ [26]	+	–	–	–	–
Hong et al. [27]	–	–	+	+	–
Klein et al. [23]	+	+	+	+	–
Kupferberg et al. ^a^ [28]	+	–	–	–	–
Oerbeck et al. [24]	+	+	+	+	+
Ooi et al. ^a^ [13]	+	+	+	+	–
Siroky et al. ^a^ [29]	+	–	–	–	–
Tan et al. [14]	+	+	+	+	–
Total # of articles analyzed	10	6	8	8	4

^a^The total scores were presented; thus, we calculated an average score and estimated standard deviation dividing the total score and standard deviation for each subsection by the number of items

### Meta-analysis of the total SMQ score

**Fig. 2:**
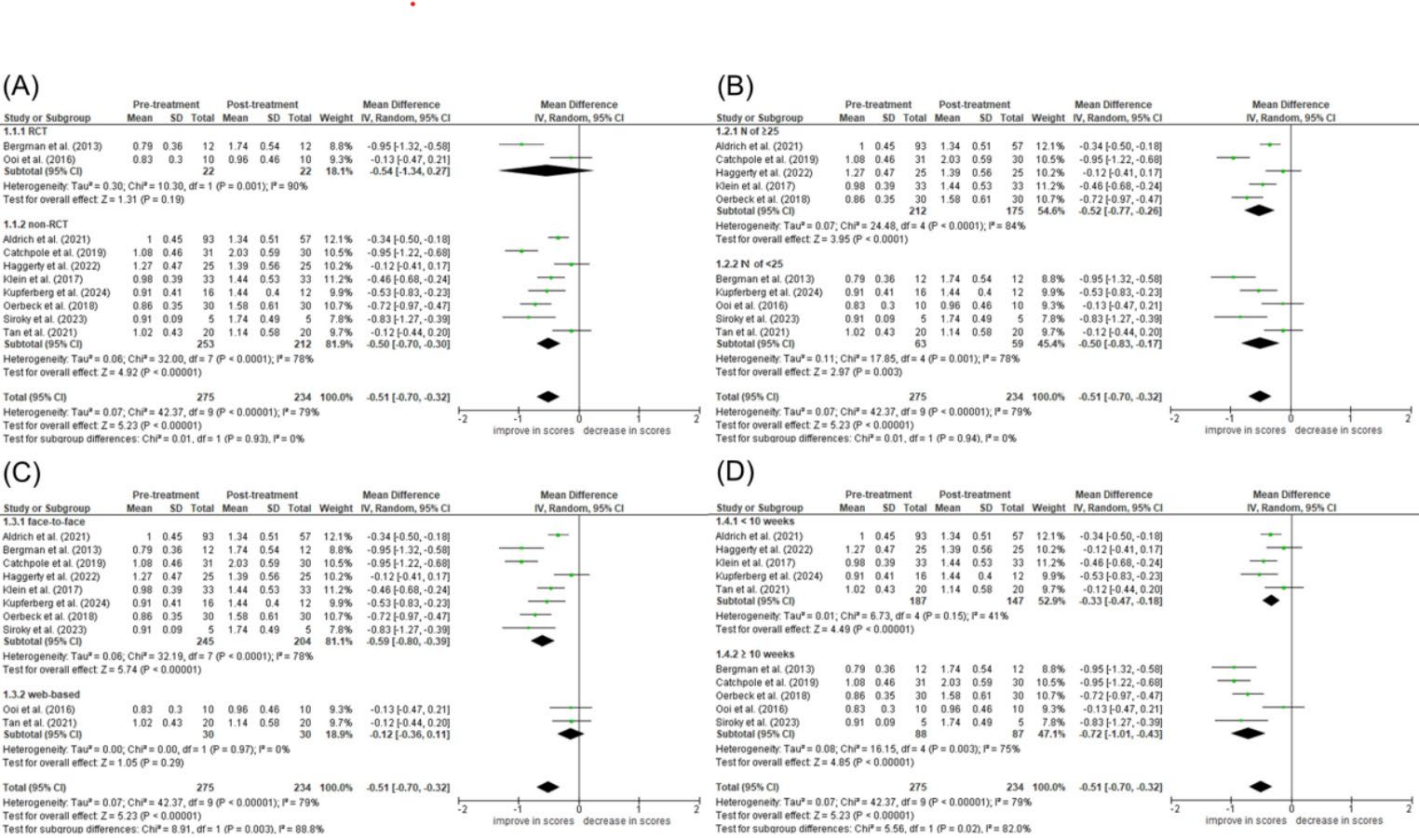
Sensitivity analysis of the included articles

**Fig. 3:**
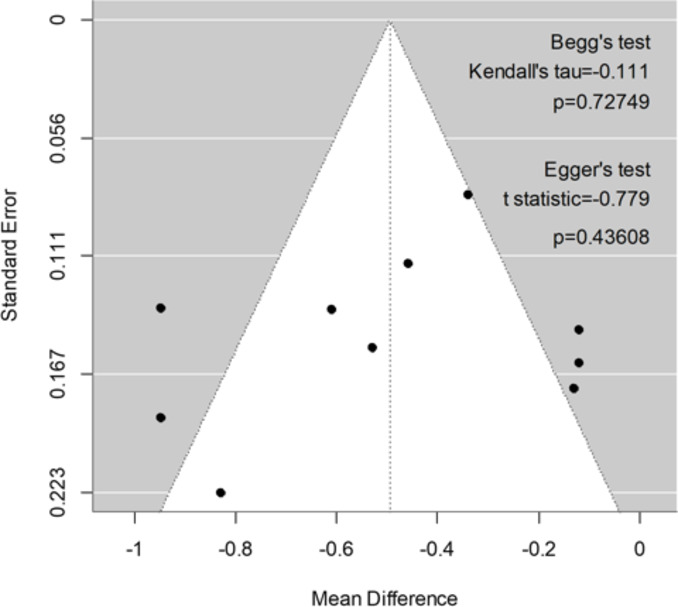
Assessment of publication bias using a funnel plot

Ten studies were pooled using the mean difference and 95% CI for the total SMQ score. The weighted mean difference scores between pre- and post-treatment of the average score of all SMQ items was − 0.51 [− 0.32– − 0.70], indicating a 0.51 improvement in the total SMQ score owing to SM treatment. The standardized effect size was large (Hedges’ adjusted g = 1.00 [0.62–1.39]). To identify whether subgroups influenced treatment efficacy, sensitivity analyses were performed on study design, sample size, intervention strategy, or treatment period, showing whether these subgroups affect the SMQ total weighted mean difference score (Fig. 2A, B, C and D). Study design and sample size did not show statistical significances (*χ2*(1) = 0.01, *p* =.0.93–94; Fig. 2A and B), indicating no influence on SMQ weighted mean difference scores. However, the intervention strategy, that is, face-to-face or web-based treatment, showed a significant difference (*χ2*(1) = 8.91, *p* <.0.001; Fig. 2C), and the 95% CI of the web-based treatment subgroup overlapped zero (the weighted mean difference score was − 0.12 [− 0.36–0.11] with a small effect size (Hedges’ adjusted g = 0.26 [− 0.25–0.77]). The treatment period subgroup also showed a significant difference (*χ2*(1) = 5.56, *p* =.0.02; Fig. 2D). Studies with longer treatment periods (≥ 10 weeks) have superior treatment efficacy, but the 95% CI of both groups did not overlap the zero. Therefore, web-based treatment is considered to have a different treatment effect from other studies; hence, we evaluated the efficacy of SM interventions in face-to-face treatment studies. The weighted mean difference score was then − 0.59 [-0.39– -0.80] with a large effect size (Hedges’ adjusted g = 1.18 [0.76–1.60]) in the face-to-face treatment. In other words, the treatment of SM improved the SMQ score by 0.59. Regarding the imprecision assessment, the overall outcome of subgroups of face-to-face treatment (eight articles) did not overlap with zero, confirming SM’s significant improvement. There was heterogeneity in those articles, but it did not impact the overall effect of the SMQ score. A funnel plot is shown in Fig. 3. No obvious publication bias was detected by inspecting the plot or statistical tests of no correlation using rank correlation (Begg’s test; *p* =.0.73) or regression model (Egger’s test; *p* = 0.44).

.

**Fig. 4:**
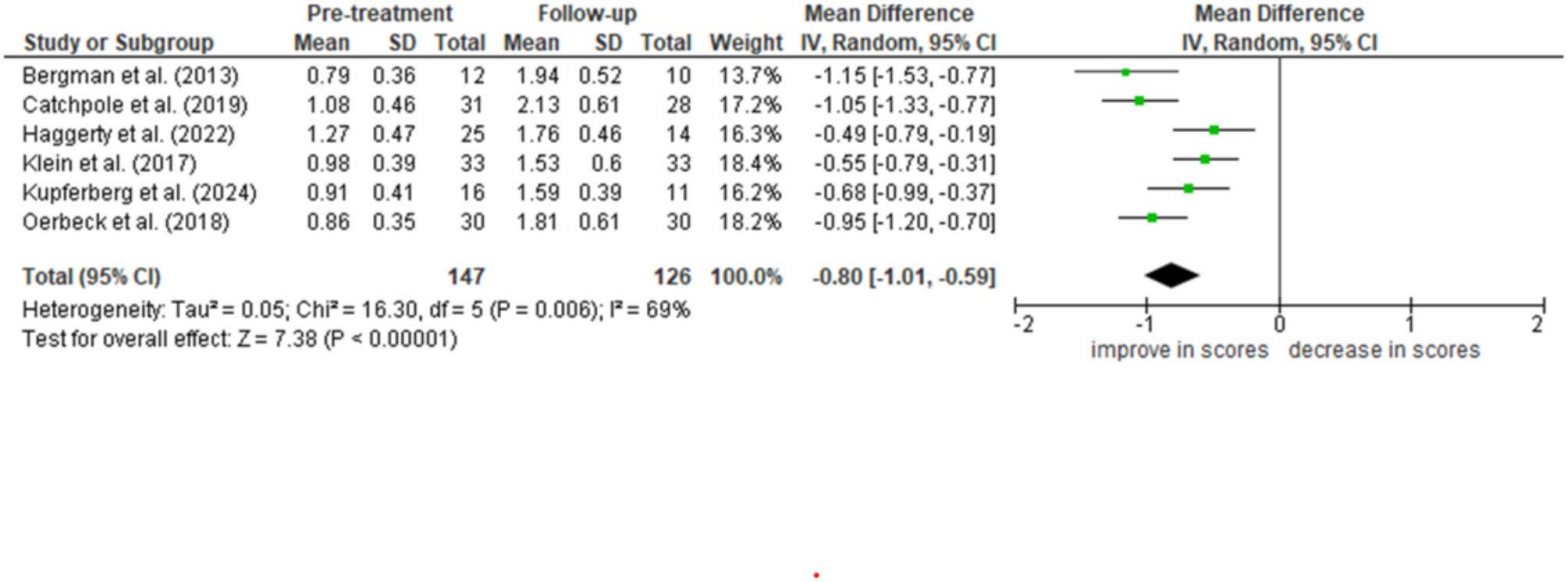
Forest plot of SMQ subsections

In the follow-up analysis, we used only SMQ total scores because of the limited number of studies that used SMQ subsection and SSQ scores. When comparing pre-treatment and follow-up data, we used the first follow-up period if a study had multiple follow-up periods, and used only face-to-face treatment studies because web-based interventions have a lesser treatment effect. The weighted mean difference score was calculated to be − 0.80 [-0.59– − 1.01] with a large effect size (Hedges’ adjusted g = 1.59 [1.17–2.02]) (Fig. 4), indicating a significant treatment effect even in the follow-up period. The significance was further verified by a sensitivity analysis, and no significant difference was found when the second follow-up period data were used (two articles contained multiple follow-up periods) and when the web-based intervention data was added and considered along with face-to-face intervention data. The weighted mean score of the second follow-up period improved further compared to the post-treatment period, but did not differ significantly (*χ2*(1) = 1.92, *p* = 0.17*).*

###  Meta-analysis of the subsections of the SMQ and total score of the SSQ

**Fig. 5:**
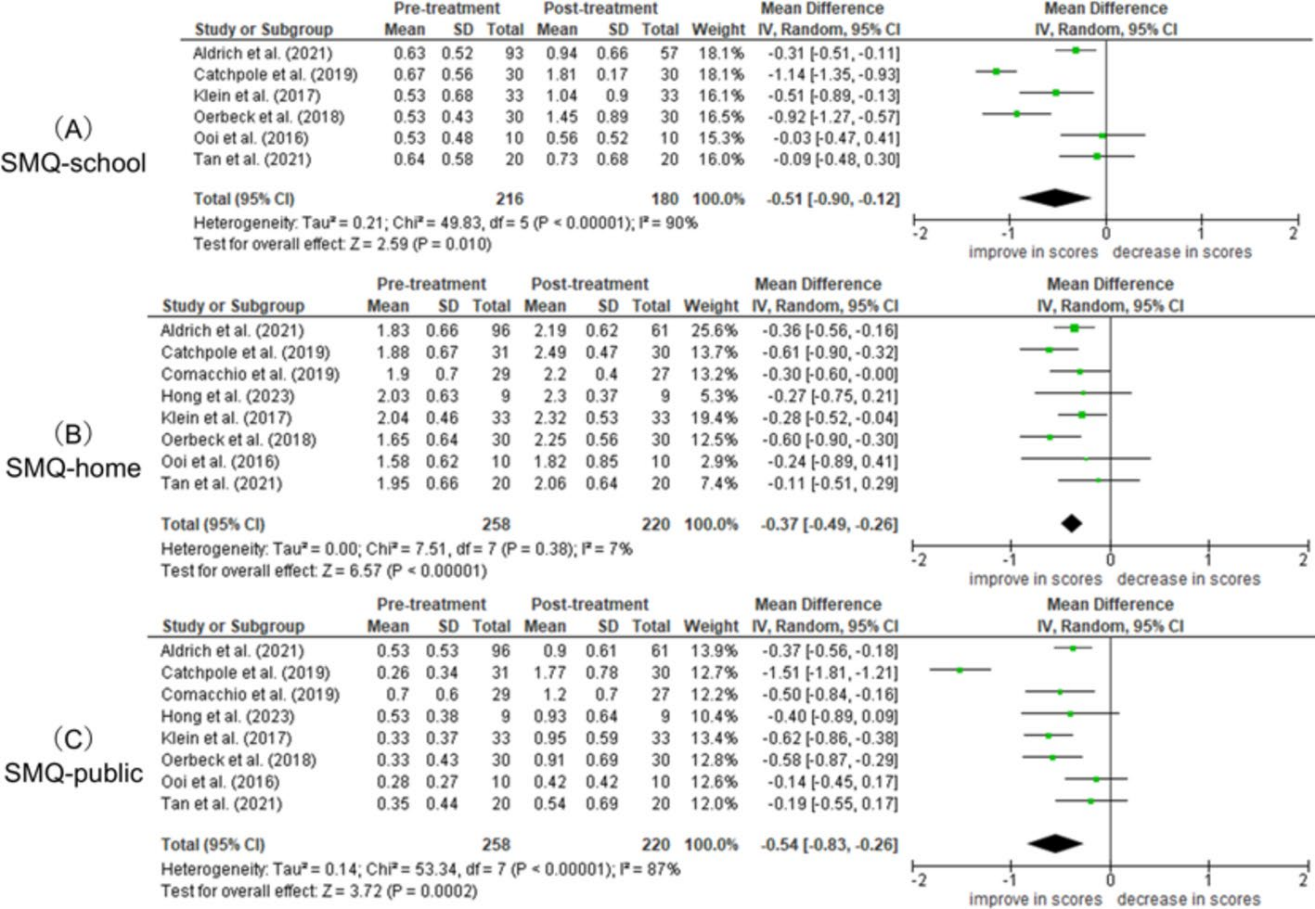
Forest plot of follow-up efficacy of total SMQ score

**Fig. 6:**
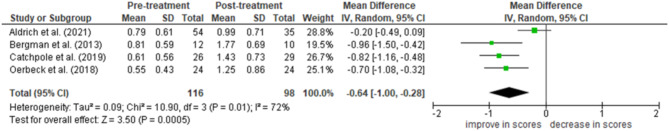
Forest plot of SSQ

As a secondary outcome, the weighted mean difference scores of the three subsections (school, home, and public) of the SMQ are shown in Fig. 5. A total of six, eight, and eight studies were analyzed in the SMQ school, home, and public scores, respectively. The weighted mean difference scores were − 0.51 [− 0.12– − 0.90] (Hedges’ adjusted g of 0.89 [0.25–1.54]) in SMQ-school (Fig. 5A), − 0.37 [− 0.26– − 0.49] (Hedges’ adjusted g of 0.61 [0.43–0.80]) in SMQ-home (Fig. 5B), and − 0.54 [-0.26– − 0.83] (Hedges’ adjusted g of 0.95 [0.53–1.38]) in SMQ-public (Fig. 5C). Additionally, SSQ was available in only four articles, and the weighted mean difference score of SSQ was − 0.64 [-0.28– -1.00] (Hedges’ adjusted g of 1.00 [0.42–1.58]) (Fig. 6). The significant efficacy of SM treatment was illustrated in all secondary outcomes.

### RoB assessment

**Fig. 7:**
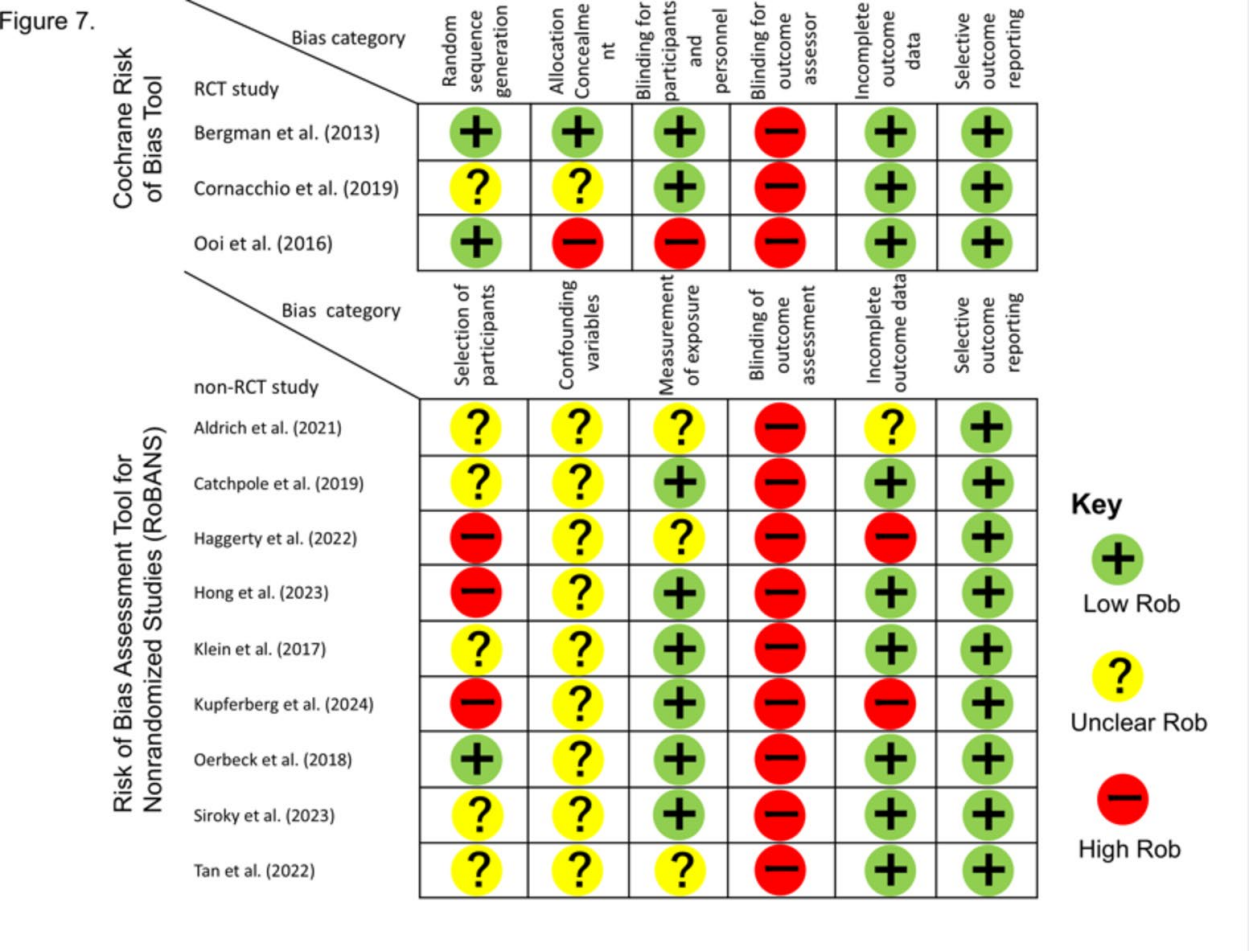
Risk of bias assessment

The results of the RoB assessment are shown in Fig. 7. The point-by-point inter-rater agreement of the assessment was 0.71. We reached a consensus on all disagreements through discussion.

In the RCT study, two articles blinded participants and personnel, and RoB was judged as “low.” In the before-after study, four articles had an “unclear” selection of participants since they did not adequately describe whether participants were recruited consecutively. In the non-RCT study, measurement of exposure, that is, the diagnoses of SM, was obtained by subjective structured interviews (The Anxiety Disorders Interview Schedule [35]) in six articles, and the performance bias was judged as “low.” As the primary outcome of our review is SMQ, a parent-reported questionnaire that could not be blinded, the blinding for outcome assessor/assessment (detection bias) were judged as “high” for all articles. As attrition bias is caused by the inadequate handling of incomplete outcome data, most articles report the number of participants before and after the study and describe that attrition is irrelevant to the study outcomes; for example, missing data is from the non-treatment group. In such cases, incomplete outcome data were judged as “low.” The selective outcome reporting bias was judged as “low.” Most articles defined clinical outcomes before the intervention and described them in the discussion. The “high” detection bias across all articles can be attributed to the unblinding feature of this questionnaire. For other items, all articles were considered to be of adequate quality and were retained in the meta-analysis.

## Discussion

This study conducted a meta-analysis of research articles that measured SMQ and SSQ scores as clinical outcomes of SM behavioral interventions. Twelve articles were eligible for our meta-analysis.

### Treatment efficacy measured by SMQ

Our meta-analysis revealed that SM treatments improved the total SMQ scores by 0.51 [0.32–0.70] (positive and negative values are described in reverse for readability). It should be noted that there were potential subgroups, as heterogeneity was observed in the included studies. In the sensitivity analysis, RCT and before-after or multiple baseline designs showed similar treatment efficacy, suggesting that before-after or multiple baseline designs can improve SM symptoms as effectively as RCTs. Similarly, studies based on sample size also did not differ significantly. Studies with treatment periods of 10 or more weeks showed greater improvement compared to studies with less than 10 weeks. As the confidence intervals for both subgroups did not overlap zero, it is interpreted that a significant treatment effect was obtained in both groups. By contrast, regarding treatment modalities, web-based treatments showed significantly less improvement in the weighted mean difference scores of the total SMQ scores compared with face-to-face treatments, which improved the total SMQ scores by 0.59 [0.39–0.80]. In one RCT study [13], the group that engaged in a web-based CBT program showed no improvement in speech frequency or anxiety symptoms compared with the control group that interacted with a therapist while playing their favorite computer game. Low improvement in web-based treatments may be because of a lack of parental psychoeducation, insufficient parent and teacher involvement, exclusion of children with a history of pharmacological [13] or other treatment experiences [13, 14], or non-individualized virtual reality exposure scenarios [14]. Although these limitations could not be addressed by face-to-face treatments, this still suggests that face-to-face interactions with a therapist may be more effective in improving SM symptoms. Even after removing two articles on web-based treatment, there was still heterogeneity in the eight articles on face-to-face treatment. Potential comparison groups that were not pre-specified may still affect the weighted mean difference scores of the SMQ total. For example, the studies of Bergman et al. [11] and Catchpole et al. [21] showed greater total SMQ score improvements, and the mean age was relatively lower compared to those in other studies (see Table 1). Oerbeck et al. [32] also found greater improvement in younger children with SM. Therefore, a greater treatment effect could be obtained if the participants’ ages were lower. As the intervention program may differ with age, controlling the intervention program or making a subgroup analysis may be necessary. Further clinical studies must verify the treatment efficacy of different groups and interventions.

The strength of our meta-analysis is that we primarily combined one type of outcome: the average score of all the SMQ items. The value of the weighted overall outcome was specific and easy to interpret. As the SMQ consisted of 17 items, its scores were expected to increase by 8.67 [5.44–11.9] points after SM treatment. A major issue with SM is comorbid anxiety symptoms, and studies measuring SMQ and anxiety often report significant improvement in anxiety and SMQ scores [11, 21, 22, 26, 29]. While anxiety scales used across studies varied, the observed improvements in anxiety and SMQ scores suggest some degree of constructive validity for the SMQ. As previously pointed out, defining what constitutes a clinically meaningful symptom improvement can be challenging [24], as no cut-off values for SMQ scores have been established. Therefore, the synthesized SMQ change values in this study may serve as a clinically meaningful benchmark for SM treatment. In the before-after SM intervention of Catchpole [21], improvement in speaking behavior was classified under three groups: non-responders [SMQ change range of −0.28–0.06], modest responders [SMQ change range of 0.41–0.85], and robust responders [SMQ change range of 0.98–2.12]. The lower 95% CI of face-to-face intervention in our meta-analysis was also 0.41, indicating moderate response [21]. Further, we confirm that the total SMQ score maintained in the same range for the post-treatment period or increased further in the post-treatment; the latter was not significant. Clinicians could refer to our results to better reflect on cases where the treatment is less effective.

When comparing the SM intervention effect sizes, Hedges’ adjusted g, which means the standardized mean difference that adjusts for bias when the sample size is small, was large (g = 1.18 [0.76–1.60] for the face-to-face intervention. In previous meta-analyses, which synthesized treatment effects from multiple RCT outcomes, Steains et al. [8] showed that there was a large effect size for the psychological intervention in five studies (g = 0.87 [0.58–1.16]), while Hipolito et al. [9] showed the same in three studies with waitlist controls (g = 1.06 [0.57–1.56]) and two studies with active controls (g = 0.55 [−0.47–1.57]). Note that the same RCTs were included in respective meta-analyses. Though it should be noted that the mean difference was obtained by comparing pre- and post- treatments scores within a treatment group in our study, not by comparison with a control group, i.e., RCT design, our meta-analysis result is interpreted as comparable to those of previous meta-analyses. Our results indicate that SMQ is comparable to other outcomes in measuring the efficacy of behavioral SM treatment, with concurrent validity and responsiveness. Given the extensive use of SMQ in many studies [4], our findings confirm the efficacy of using SMQ.

### Subsections of SMQ and SSQ

In the SMQ subsections, the treatment effect was significantly improved in public and school, but there was less improvement in the home. This could be because, children with SM usually speak at home, unlike in other social situations, as per the DSM-5 definition [1]. The average scores of SMQ-school or SMQ-public in pre-treatment were less than 1.0 in all articles, which means “never” talk (scored 0) or “seldom” talk (scored 1) in each situation. However, SMQ-home in pre-treatment is around 2.0, which means “often” talk (scored 2) in each situation (Fig. 5B). Another possible reason for low improvement in SMQ-home scores is that improvement is more likely in situations where the intervention was carried out. Although a few articles included home as an intervention setting, school or public environments were the most common treatment settings. Therefore, SMQ-school or SMQ-public could sensitively reflect the treatment efficacy of SM. The SSQ was included in only four articles and had a substantial treatment efficacy (weighted mean difference: 0.64 [0.28–1.00]). While SMQ is a parent-reported questionnaire, SSQ is used in a school setting and completed by a teacher. Teachers observing SM children in school can accurately reflect real-life situations using the SSQ, as SM symptoms are typically observable at school. As described above, the effects of SM treatment are more likely to appear in the school and public subsections of the SMQ, and the effects are even more pronounced on the SSQ. Thus, future assessments of treatment effects should include reporting of SSQ scores along with scores on the SMQ subsections.

### Limitations and future implications

First, as we included study designs without control groups, the pre- and post-treatment scores were synthesized even for RCT design studies. Therefore, confounding factors other than treatment may be reflected in changes in SMQ scores. Further accumulation of SM intervention studies in RCT designs is needed to address this limitation. The effect size obtained in our meta-analysis would be helpful for power analysis to estimate the sample size of a future behavioral treatment study of SM. Second, the SMQ and SSQ were administered by parents and teachers; therefore, evaluators’ bias could not be eliminated. However, owing to their simplicity, the questions can be easily used in clinical settings as indicators of treatment effect. Third, more than half of the studies reported only specific subsections of the SMQ or a portion of the SSQ, making treatment effect comparisons across studies challenging. Therefore, future studies should cumulatively update the clinical efficacy of SM treatment by including all subsections of the SMQ and the SSQ as measures of treatment effects to better examine the specific impacts of each treatment. Fourth, the representativeness of the included studies may be limited, as most studies were from North America, with a few studies from other countries such as Europe or Asia. Therefore, cultural and ethnic biases may be reflected in our meta-analysis. As mentioned earlier in the discussion, further verification of potential subgroups related to the heterogeneity of scores is needed, we expect that more reports in various populations or settings would clarify subgroup differences. For future updates of the meta-analysis, this study did not register the study protocols in advance with PROSPERO or other organizations, but it would be necessary to register the protocols in advance to increase the transparency of the meta-analysis.

## Conclusions

Our meta-analysis synthesized the existing evidence on the SMQ and SSQ as measures of the clinical outcomes of SM. Although the apparent treatment effect is observed only in the face-to-face treatment and other potential subgroups exist, we revealed the efficacy of behavioral interventions that used the SMQ or SSQ.

## Electronic supplementary material

Supplementary Material 1.

## Data Availability

No datasets were generated or analysed during the current study.

## Abbreviations

CBCL: Child behavior checklist
CI: confidence intervals
DSM: Diagnostic and statistical manual of mental disorders
PCIT: Parent child interaction therapy
PRISMA: Preferred reporting items for systematic reviews and meta-analysis
RCT: Randomized controlled trial
RoB: Risk of bias
RoBANS: Risk of bias assessment tool for nonrandomised studies
SM: Selective mutism
SNAP: Strong narrative assessment procedure
SMQ: Selective mutism questionnaire
SSQ: School speech questionnaire

## References

[1] American Psychiatric Association. Diagnostic and statistical manual of mental disorders: DSM-5. 5th ed. American Psychiatric Association; 2013.

[2] Muris P, Ollendick TH. Selective mutism and its relations to social anxiety disorder and autism spectrum disorder. Clin Child Fam Psychol Rev. 2021;24:294–325. 10.1007/s10567-020-00342-0. 33462750 10.1007/s10567-020-00342-0 PMC8131304

[3] A_Østergaard KR. Treatment of selective mutism based on cognitive behavioural therapy, psychopharmacology and combination therapy–a systematic review. Nord J Psychiatry. 2018;72(4):240–50. 10.1080/08039488.2018.1439530. 29447060 10.1080/08039488.2018.1439530

[4] Rodrigues Pereira C, Ensink JBM, Güldner MG, Lindauer RJL, De Jonge MV, Utens EMWJ. Diagnosing selective mutism: A critical review of measures for clinical practice and research. Eur Child Adolesc Psychiatry. 2023;32:1821–39. 10.1007/s00787-021-01907-2. 34853909 10.1007/s00787-021-01907-2 PMC10533577

[5] Bergman RL, Keller ML, Piacentini J, Bergman AJ. The development and psychometric properties of the selective mutism questionnaire. J Clin Child Adolesc Psychol. 2008;37:456–64. 10.1080/15374410801955805. 18470781 10.1080/15374410801955805

[6] Bergman RL, Piacentini J, McCracken JT. Prevalence and description of selective mutism in a school-based sample. J Am Acad Child Adolesc Psychiatry. 2002;41:938–46. 10.1097/00004583-200208000-00012. 12162629 10.1097/00004583-200208000-00012

[7] Oerbeck B, Overgaard KR, Bergman RL, Pripp AH, Kristensen H. The selective mutism questionnaire: data from typically developing children and children with selective mutism. Clin Child Psychol Psychiatry. 2020;25:754–65. 10.1177/1359104520914695. 32281879 10.1177/1359104520914695 PMC7528533

[8] Steains SY, Malouff JM, Schutte NS. Efficacy of psychological interventions for selective mutism in children: A meta-analysis of randomized controlled trials. Child Care Health Dev. 2021;47:771–81. 10.1111/cch.12895. 34265102 10.1111/cch.12895

[9] Hipolito G, Pagnamenta E, Stacey H, Wright E, Joffe V, Murayama K, et al. A systematic review and meta-analysis of nonpharmacological interventions for children and adolescents with selective mutism. JCPP Adv. 2023;3:e12166. 10.1002/jcv2.12166. 37720585 10.1002/jcv2.12166 PMC10501694

[10] Strong CJ, Mayer M, Mayer M. The strong narrative assessment procedure (SNAP). Thinking; 1998.

[11] Bergman RL, Gonzalez A, Piacentini J, Keller ML. Integrated behavior therapy for selective mutism: A randomized controlled pilot study. Behav Res Ther. 2013;51:680–9. 10.1016/j.brat.2013.07.003. 23933108 10.1016/j.brat.2013.07.003

[12] Oerbeck B, Stein MB, Wentzel-Larsen T, Langsrud Ø, Kristensen H. A randomized controlled trial of a home and school-based intervention for selective mutism– defocused communication and behavioural techniques. Child Adolesc Ment Health. 2014;19:192–8. 10.1111/camh.12045. 32878377 10.1111/camh.12045

[13] Ooi YP, Sung SC, Raja M, Kwan C, Koh JBK, Fung D. Web-based CBT for the treatment of selective mutism: results from a pilot randomized controlled trial in Singapore. J Speech Pathol Ther. 2016;1:112.

[14] Tan YR, Ooi YP, Ang RP, Goh DH, Kwan C, Fung DS, et al. Feasibility trial of virtual reality exposure therapy for selective mutism. Clin Child Psychol Psychiatry. 2022;27:351–68. 10.1177/13591045211056920. 34866415 10.1177/13591045211056920

[15] Page MJ, Moher D, Bossuyt PM, Boutron I, Hoffmann TC, Mulrow CD, et al. PRISMA 2020 explanation and elaboration: updated guidance and exemplars for reporting systematic reviews. BMJ. 2021;372:n160. 10.1136/bmj.n160. 33781993 10.1136/bmj.n160 PMC8005925

[16] Higgins JPT, Altman DG, Gøtzsche PC, Jüni P, Moher D, Oxman AD, et al. The Cochrane collaboration’s tool for assessing risk of bias in randomised trials. BMJ. 2011;343:d5928. 10.1136/bmj.d5928. 22008217 10.1136/bmj.d5928 PMC3196245

[17] Kim SY, Park JE, Lee YJ, Seo HJ, Sheen SS, Hahn S, et al. Testing a tool for assessing the risk of bias for nonrandomized studies showed moderate reliability and promising validity. J Clin Epidemiol. 2013;66:408–14. 10.1016/j.jclinepi.2012.09.016. 23337781 10.1016/j.jclinepi.2012.09.016

[18] The Cochrane Collaboration. Review manager (RevMan). Version 5.4.1 [Computer software]. Copenhagen: The Nordic Cochrane Centre; 2020.

[19] Higgins JPT, Thompson SG, Deeks JJ, Altman DG. Measuring inconsistency in meta-analyses. BMJ. 2003;327:557–60. 10.1136/bmj.327.7414.557. 12958120 10.1136/bmj.327.7414.557 PMC192859

[20] R Core Team. R: A Language and Environment for Statistical Computing; 2021. Vienna, Austria: R Foundation for Statistical Computing. https://www.r-project.org/

[21] Catchpole R, Young A, Baer S, Salih T. Examining a novel, parent child interaction therapy-informed, behavioral treatment of selective mutism. J Anxiety Disord. 2019;66:102112. 10.1016/j.janxdis.2019.102112. 31351241 10.1016/j.janxdis.2019.102112

[22] Cornacchio D, Furr JM, Sanchez AL, Hong N, Feinberg LK, Tenenbaum R, et al. Intensive group behavioral treatment (IGBT) for children with selective mutism: A preliminary randomized clinical trial. J Consult Clin Psychol. 2019;87:720–33. 10.1037/ccp0000422. 31294589 10.1037/ccp0000422 PMC6629469

[23] Klein ER, Armstrong SL, Skira K, Gordon J. Social communication anxiety treatment (S-CAT) for children and families with selective mutism: A pilot study. Clin Child Psychol Psychiatry. 2017;22:90–108. 10.1177/1359104516633497. 26940121 10.1177/1359104516633497

[24] Oerbeck B, Overgaard KR, Stein MB, Pripp AH, Kristensen H. Treatment of selective mutism: A 5-year follow-up study. Eur Child Adolesc Psychiatry. 2018;27:997–1009. 10.1007/s00787-018-1110-7. 29357099 10.1007/s00787-018-1110-7 PMC6060963

[25] Landis JR, Koch GG. An application of hierarchical kappa-type statistics in the assessment of majority agreement among multiple observers. Biometrics. 1977;33:363–74. 10.2307/2529786. 884196

[26] Haggerty D, Carlson JS, Kotrba A. A pilot feasibility study of an intensive summer day camp intervention for children with selective mutism. Children. 2022;9(11):1732. 10.3390/children9111732. 36421181 10.3390/children9111732 PMC9689151

[27] Hong N, Herrera A, Furr JM, Georgiadis C, Cristello J, Heymann P, Dale CF, Heflin B, Silva K, Conroy K, Cornacchio D, Comer JS. Remote intensive group behavioral treatment for families of children with selective mutism. E Evid Based Pract Child Adolesc Ment Health. 2023;8(4):439–58. 10.1080/23794925.2022.2062688. 38155719 10.1080/23794925.2022.2062688 PMC10752620

[28] Kupferberg R, Avny S, Ortiz C. Intensive group behavioral treatment for older youth with selective mutism: an open trial. Cogn Behav Pract. 2024. 10.1016/j.cbpra.2024.08.004.

[29] Siroky AK, Carlson JS, Kotrba A. Integrated behavior therapy for exclusively anxious selective mutism: A nonconcurrent Multiple-Baseline design across five participants. Pediatr Rep. 2023;15(4):617–35. 10.3390/pediatric15040057. 37873803 10.3390/pediatric15040057 PMC10594503

[30] Aldrich JT, Blossom JB, Moss A, Ray B, Couckuyt M, Ward T, et al. Effectiveness of an eight-week multidisciplinary selective mutism treatment group. Evid Based Pract Child Adolesc Ment Health. 2023;8:105–19. 10.1080/23794925.2021.2007818.

[31] Oerbeck B, Johansen J, Lundahl K, Kristensen H. Selective mutism: A home-and kindergarten-based intervention for children 3–5 years: A pilot study. Clin Child Psychol Psychiatry. 2012;17:370–83. 10.1177/1359104511415174. 21852320 10.1177/1359104511415174

[32] Oerbeck B, Stein MB, Pripp AH, Kristensen H. Selective mutism: Follow-up study 1 year after end of treatment. Eur Child Adolesc Psychiatry. 2015;24:757–66. 10.1007/s00787-014-0620-1. 25267381 10.1007/s00787-014-0620-1 PMC4490179

[33] Achenbach TM, Rescorla LA. *Manual for the ASEBA preschool forms and profiles*, *30*. Burlington, VT: University of Vermont; 2000, Research center for children, youth, & families.

[34] Spence SH. A measure of anxiety symptoms among children. Behav Res Ther. 1998;36:545–66. 10.1016/S0005-7967(98)00034-5. 9648330 10.1016/s0005-7967(98)00034-5

[35] Silverman W, Albano A. The anxiety disorders interview schedule for children (ADIS-C/P). Psychological Corporation; 1996

