# Supplementary material for: Meta-analysis of behavioral treatments for selective mutism: findings from selective mutism questionnaire (SMQ) and school speech questionnaire (SSQ)
Source: Child Adolesc Psychiatry Ment Health. 2025 Apr 3;19:40. doi: 10.1186/s13034-025-00891-8 (PMC11969985; doi:10.1186/s13034-025-00891-8)
Supplement: Supplementary file 1 — Supplementary Material 1. [file 13034_2025_891_MOESM1_ESM.docx]

| No. | Article | Reasons for exclusion |
| --- | --- | --- |
| 1 | Casas, M. A., & Conn, B. M. (2024). Narrative exposure therapy for treating post-traumatic stress among transgender youth of color with selective mutism. Clinical Case Studies, 23(1), 63-81. | e) no SMQ data |
| 2 | Cengher, M., Clayborne, J. C., Crouch, A. E., & O’Connor, J. T. (2021). Assessment and treatment of selective mutism in a child with autism spectrum disorder. Clinical Case Studies, 20(3), 248–264. | e) no SMQ data |
| 3 | Cornacchio, D. (2019). Evaluating Intensive Group Behavioral Treatment for Children with Selective Mutism. Florida International University. | b) dissertation |
| 4 | Dogru, H., Ucuz, I., Uzun Cicek, A., & Comertoglu Arslan, S. (2023). Clinical characteristics according to sex and symptom severity in children with selective mutism: a four-center study. Nordic Journal of Psychiatry, 77(2), 158-164. | e) no SMQ data |
| 5 | Godoi, D. H., Azevedo, H., Rodrigues, C. L., Rocca, C. C. D. A., Beraldo Filho, G., Castro, L. L., Asbahr, F., & Romero, R. A. F. (2024). Preliminary impressions: Using a humanoid robot in the neuropsychological assessment of children with selective mutism. International Journal of Advanced Robotic Systems, 21(5). | e) no SMQ data |
| 6 | Jones, K., & Odell-Miller, H. (2023). A theoretical framework for the use of music therapy in the treatment of selective mutism in young children: Multiple case study research. Nordic Journal of Music Therapy, 32(1), 4–28. | e) no SMQ data |
| 7 | Kwok Kar Kin, G. (2022). Mutism and sandplay therapy: A child's silent journey. Journal of Sandplay Therapy, 31(1). | e) no SMQ data |
| 8 | Liu, S. (2021). Considerations for Adapting Parent Child Interaction Therapy (PCIT) for Chinese Americans. Pepperdine University. | b) dissertation |
| 9 | Lorenzo, N. E., Cornacchio, D., Chou, T., Kurtz, S. M., Furr, J. M., & Comer, J. S. (2021). Expanding treatment options for children with selective mutism: rationale, principles, and procedures for an intensive group behavioral treatment. Cognitive and Behavioral Practice, 28(3), 379–392. | a) review |
| 10 | Ludlow, A. K., Osborne, C., & Keville, S. (2023). Selective mutism in children with and without an autism spectrum disorder: The role of sensory avoidance in mediating symptoms of social anxiety. Journal of Autism and Developmental Disorders, 53(10), 3891-3900. | g) no SM intervention |
| 11 | Ng, V. Y. (2023). Parent-based group treatment for childhood selective mutism: A pilot non-inferiority trial of supportive parenting for anxious childhood emotions. Hofstra University. | 1. b) dissertation |
| 12 | Pabis, J. M. (2023). Parent training workshop for children with selective mutism: A randomized-controlled trial. Northern Illinois University. | 1. b) dissertation |
| 13 | Rosheim, K. (2022). Learning from Tina: a case study with a selective speaker. Language Awareness, 31(2), 175–193. | e) no SMQ data |
| 14 | Shorer, M., Ben-Haim, Z., Klauzner, N., Ben-Ami, N., & Fennig, S. (2023). The Integrated Behavior Therapy for Children with Selective Mutism: Findings from an open pilot study in a naturalistic setting. Clinical Child Psychology and Psychiatry, 28(2), 465–482. | h）no statistical values |
| 15 | van Deurs, J. R., McLay, L. K., France, K. G., & Blampied, N. M. (2021). Sequential implementation of functional behavior assessment-informed treatment components for sleep disturbance in autism: A case study. Behavioral Sleep Medicine, 19(3), 333–351. | e) no SMQ data |
| 16 | Yamanaka, T., Ishida, Y, & Inoue, M. (2023). Long-term outcome of selective mutism: factors influencing the feeling of being cured. European Child & Adolescent Psychiatry, 32(11), 2209–2221. | g) no SM intervention |
